# A Low-Activity Polymorphic Variant of Human NEIL2 DNA Glycosylase

**DOI:** 10.3390/ijms23042212

**Published:** 2022-02-17

**Authors:** Zarina I. Kakhkharova, Dmitry O. Zharkov, Inga R. Grin

**Affiliations:** 1 SB RAS Institute of Chemical Biology and Fundamental Medicine, 630090 Novosibirsk, Russia; z.kakhkharova@alumni.nsu.ru; 2 Department of Natural Sciences, Novosibirsk State University, 630090 Novosibirsk, Russia

**Keywords:** DNA damage, DNA repair, DNA glycosylases, NEIL2, single-nucleotide polymorphisms, variants of unknown significance

## Abstract

Human NEIL2 DNA glycosylase (hNEIL2) is a base excision repair protein that removes oxidative lesions from DNA. A distinctive feature of hNEIL2 is its preference for the lesions in bubbles and other non-canonical DNA structures. Although a number of associations of polymorphisms in the *hNEIL2* gene were reported, there is little data on the functionality of the encoded protein variants, as follows: only hNEIL2 R103Q was described as unaffected, and R257L, as less proficient in supporting the repair in a reconstituted system. Here, we report the biochemical characterization of two hNEIL2 variants found as polymorphisms in the general population, R103W and P304T. Arg103 is located in a long disordered segment within the N-terminal domain of hNEIL2, while Pro304 occupies a position in the β-turn of the DNA-binding zinc finger motif. Similar to the wild-type protein, both of the variants could catalyze base excision and nick DNA by β-elimination but demonstrated a lower affinity for DNA. Steady-state kinetics indicates that the P304T variant has its catalytic efficiency (in terms of *k*_cat_/*K*_M_) reduced ~5-fold compared with the wild-type hNEIL2, whereas the R103W enzyme is much less affected. The P304T variant was also less proficient than the wild-type, or R103W hNEIL2, in the removal of damaged bases from single-stranded and bubble-containing DNA. Overall, hNEIL2 P304T could be worthy of a detailed epidemiological analysis as a possible cancer risk modifier.

## 1. Introduction

DNA repair enzymes play a central role in the protection of living cells from genotoxic insults. Base excision repair (BER), one of the several known DNA repair pathways, safeguards the genome from a variety of small non-bulky lesions, which arise mainly through deamination, oxidation, alkylation, and base loss [1,2,3]. BER is initiated by excision of damaged bases by one of a group of enzymes called DNA glycosylases, which recognize the lesions and hydrolyze their *N*-glycosidic bond [1,4]. Most DNA glycosylases belong to the following three major structural superfamilies that are characterized by the presence of conserved structural elements: the α/β-fold, the helix–hairpin–helix motif, or the helix–two-turn–helix (H2TH) motif [5,6].

At present, eleven DNA glycosylases have been characterized from human cells [2,7]. Three of them—NEIL1, NEIL2, and NEIL3—are H2TH proteins. They are homologous to *E. coli* endonuclease VIII (Nei) and formamidopyrimidine-DNA glycosylase (Fpg), two enzymes participating in the repair of oxidative DNA damage. The roles of Nei and Fpg are different in the following ways: while Fpg is specific for oxidized purines, mostly 8-oxoguanine and formamidopyrimidines, Nei excises a number of oxidized pyrimidines. However, human cells also possess two other enzymes, 8-oxoguanine–DNA glycosylase (OGG1) and endonuclease III-like protein (NTHL1), which efficiently excise oxidized purines and pyrimidines, respectively. Thus, the functions of NEIL proteins are not clear at present, and many specialized roles had been proposed for these BER components.

Of the three NEIL proteins, NEIL2 remains the most enigmatic. In its substrate specificity, it is much closer to Nei than to Fpg. It efficiently removes oxidized pyrimidine bases, such as 5-hydroxyuracil (OHU) and 5,6-dihydrouracil (DHU), as well as spiroiminodihydantoin and 5-guanidinohydantoin, which are the products of further oxidation of 8-oxoG [8,9,10,11]. As the specificity of NEIL2 overlaps with that of other human DNA glycosylases (NEIL1, NTHL1), the question of whether it has a unique role in the cell is not solved. Evidence points to the importance of NEIL2 for the repair of lesions in DNA structures other than the canonical B-DNA. A characteristic feature of NEIL2, which distinguishes it from other DNA glycosylases, is its ability to process DNA substrates containing lesions in bubbles, double-stranded but non-complementary structures 5–20 nt long, as well as in D-loops and R-loops [11,12]. It is assumed that this ability may reflect the role of NEIL2 in the repair of damaged bases that are located in partially unwound DNA, such as transcription bubbles or replication forks. It should be noted, however, that the absence of a correctly positioned DNA template can facilitate DNA ligation without the incorporation of a correct dNMP, which leads to a single nucleotide deletion [13]; therefore, the biological significance of the activity towards bubble substrates remains unclear.

**Figure 1:**
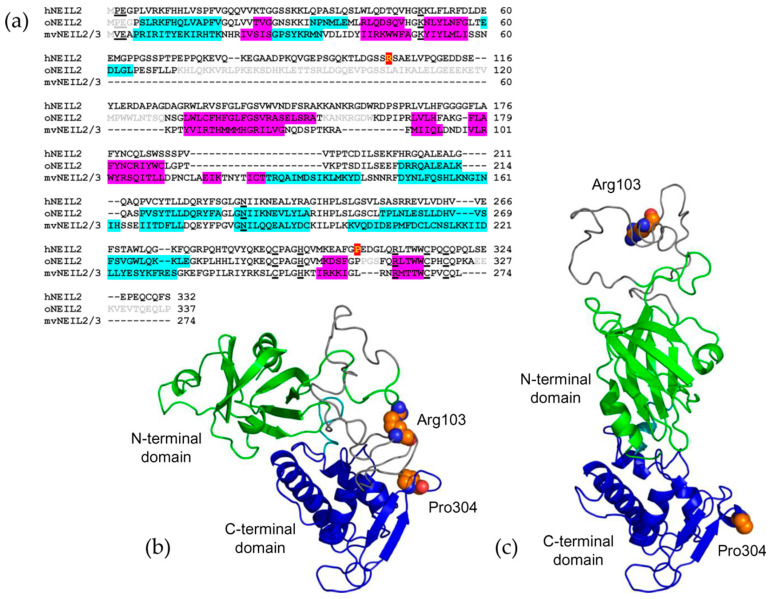
Structural models of hNEIL2. (**a**) Alignment of hNEIL2, oNEIL2, and mvNEIL2/3. α-Helices in the oNEIL2 and mvNEIL2/3 structures are highlighted cyan, β-strands are shown in magenta. The residues missing from the X-ray structures are shown in grey. The key residues participating in catalysis, DNA binding, and Zn^2+^ ion coordination are underlined. Arg103 and Pro304 in the human sequence are highlighted red. (**b**), Homology model of the closed conformation of hNEIL2 based on the mvNEIL2/3 template (4MB7 [15]). (**c**) Homology model of the open conformation of hNEIL2 based on the oNEIL2 template (6VJI [14]). In (**b**,**c**), the N-terminal domain of hNEIL2 is shown in green, the C-terminal domain, in blue, the disordered loop, in grey, and the interdomain linker is in turquoise.

The structure of human NEIL2 (hNEIL2) has not been solved so far, and the closest homologs with the known structure are NEIL2 from short-tailed grey opossum (*Monodelphis domestica*, oNEIL2; 6VJI [14]), and a more distant NEIL2/3 from *Acanthamoeba poliphaga* mimivirus (mvNEIL2/3; 4MB7, [15]) (Figure 1). The structures reveal a two-domain, organization, consisting of the N-terminal β-sandwich domain starting with an α-helix that carries the catalytic residues Pro2 and Glu3, and the C-terminal predominantly helical domain containing the H2TH motif and a β_2_ zinc finger. Unique among other H2TH DNA glycosylases, the N-terminal domain of NEIL2 in vertebrate species (but not mvNEIL2/3) possesses a long disordered loop of unknown function inserted between β3 and β4 strands. The structures of oNEIL2, mvNEIL2/3, and other H2TH DNA glycosylases suggest that these proteins may exist in two conformations, a closed one that is established after DNA binding with a fully assembled active site, and an open one with the two domains swung apart, evident in some X-ray structures of free H2TH proteins (Figure 1). Recently, the solution structure and dynamics of free hNEIL2 was assessed using hydrogen–deuterium exchange mass spectrometry, confirming the predominantly open conformation and the disorder in the N-terminal domain loop insert [16].

hNEIL2 has recently attracted attention because of its association with cancer development and progression and inflammatory response. In a study of copy number variations collected in the Catalogue of Somatic Mutations in Cancer (COSMIC) [17], the *hNEIL2* gene was found to be the gene that was most frequently lost in tumors in general, and this loss was correlated with the decreased overall survival and disease-free survival in several tumor types [18]. One particular SNP, rs1466785, located 5′ to the *hNEIL2* coding part, is strongly associated with increased cancer risk in BRCA2 mutation carriers [19] and decreased blood triglyceride levels [20], while a nearby rs804271 polymorphism increases gene expression, increases DNA damage at telomeres in *BRCA1* or *BRCA2* mutation carriers, and was classified as a cancer risk modifier [21]. The reported associations of polymorphisms in the non-coding parts of *hNEIL2* include the following: risk of squamous cell oral and oropharynx carcinoma [22], testicular germ cell tumors [23], gastric cancer [24], cervical squamous cell carcinomas and intraepithelial neoplasias [25] and breast cancer [26], decreased progression-free survival in colorectal cancer patients [27], non-small cell lung cancer patients treated with cisplatin [28] and breast cancer [26], and recurrence in BCG-treated bladder cancer [29]. Also, non-coding *hNEIL2* polymorphisms showed associations with addiction and behavioral disinhibition [30], susceptibility to age-related cataracts [31], and increased chromosome aberrations induced by tobacco nitrosamines [32]. *Neil2*^−/−^ mice are viable but show an increased production of pro-inflammatory cytokines upon bacterial infection or challenge with lipopolysaccharide [33,34,35]. Other special roles of NEIL2 could be manifested in specific cells or tissues or at specific developmental stages. It has been shown that the expression of the *Neil2* gene in the rat embryo brain begins at least at E16 and increases with time [36]. In adult mice, *Neil2* is highly expressed in skeletal muscle and testes, and at an intermediate level, in the brain and heart [8].

As a member of the H2TH superfamily, hNEIL2 possesses a similar mechanism of DNA binding and catalysis as Fpg, Nei, and NEIL1. In particular, it has been shown that Pro1 is the active nucleophile in the base excision reaction, and that the conserved residues Lys50 and Arg310 interacting with the DNA backbone near the lesion, as well as the intact structure of the zinc finger, are required for efficient DNA binding by hNEIL2 [12,37,38]. In contrast, naturally occurring protein variants, such as polymorphisms or cancer somatic mutants, are much less predictable in their functionality, and the biochemical properties of the natural hNEIL2 protein variants have been barely studied so far. Two non-synonymous missense polymorphic variants that are often observed in lung and colorectal cancer, R103Q and R257L, have been biochemically characterized, the latter demonstrating a decline in its ability to interact with other BER proteins and support the repair in a reconstituted system [39] (please note that in this paper we number the protein residues starting from Met1 in order to keep in line with the literature on hNEIL2 polymorphisms, yet in some papers concerned with the structure and mechanism the numeration start from Pro1, which is Pro2 in the preprotein but becomes the N-terminal residue in the mature polypeptide after the removal of Met1). Epidemiological studies point to the R257L allele as a risk factor for lung and cervical cancers [25,39]. Here we report the characterization of two polymorphic hNEIL2 variants of uncertain significance, R103W and P304T.

## 2. Results

### 2.1. Non-Synonymous Polymorphisms in hNEIL2

**Figure 2:**
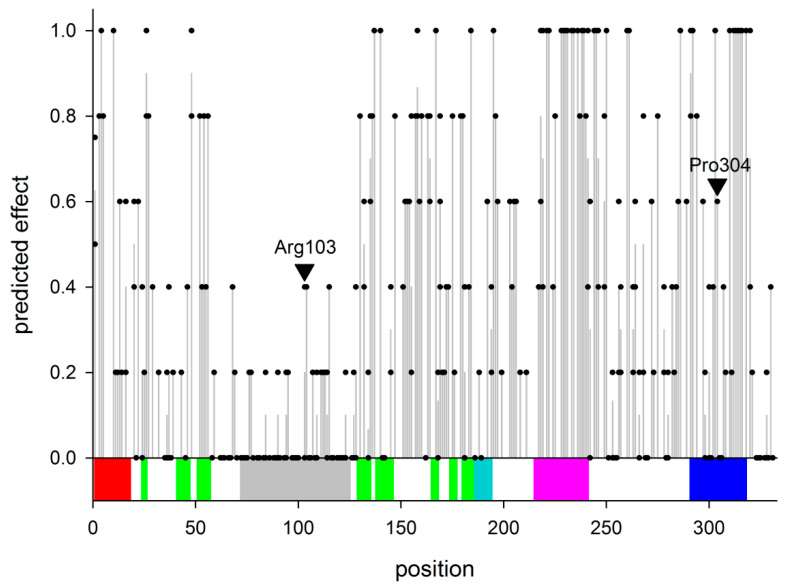
Predicted effect of mutations in hNEIL2. Black dots indicate the effect of any given variant at a particular position (Table S1). Grey bars indicate the average effect of all known polymorphisms at a particular position. Arrowheads mark the positions of R103W and P304T variants. The colored bar below corresponds to the main elements of the hNEIL2 structure as inferred based on sequence homology with oNEIL2 and mvNEIL2/3: red, the N-terminal α-helix carrying the catalytic Pro and Glu; green, β-sandwich in the N-terminal domain; grey, the disordered loop; turquoise, the interdomain linker; magenta, the H2TH motif; blue, the zinc finger.

At the beginning of the study, the dbSNP database contained 303 non-synonymous, single amino acid substitution polymorphisms in the *hNEIL2* gene (Table S1). In order to analyze the possible effect of the polymorphisms on the functions of hNEIL2, we have employed the following five prediction algorithms with different underlying classification principles: FATHMM [40], Mutation Assessor [41], MutationTaster [42], PolyPhen-2 [43], and Provean [44]. All of them, except Mutation Assessor, produce a binary output classifying the amino acid change as neutral/tolerated or damaging/deleterious. Mutation Assessor classifies the changes according to the expected damaging effect as neutral/low/medium/high, which for the purpose of our analysis were converted to the binary output as neutral/low vs medium/high. All five of the classifiers produced good agreement for the predictions on the whole set of mutations (Krippendorff’s α = 0.456; α = 1 indicates perfect agreement, α = 0 corresponds to the agreement not better than that expected by chance; α < 0 indicates disagreement exceeding that expected by chance). The pairwise percent agreement between the different algorithms varied between 62% and 81% (Cohen’s κ = 0.289–0.606; κ = 1 indicates perfect agreement, κ = 0 corresponds to the agreement not better than that expected by chance, κ < 0 indicates disagreement exceeding that expected by chance), also corresponding to good coincidence in the predictions. In order to obtain an integrated number for the mutation effect, we have translated the binary outputs into numbers 0 (no effect) or 1 (affected), and then averaged between the results of the five classifiers (Figure 2). The protein’s regions that were most densely populated with the highest-effect polymorphisms were the H2TH motif and the second β-strand of the zinc finger (Figure 2), which was quite expected due to the known functional importance of these structural elements in H2TH DNA glycosylases. On the contrary, most of the mutations in and adjacent to the disordered loop were predicted to have little effect on the hNEIL2 functions (Figure 2).

Of all of the polymorphic variants, we have selected two for further characterization, namely R103W (rs8191612) and P304T (rs8191666). The predicted effect of both of the substitutions is intermediate (Figure 2, arrowheads), yet they attracted our attention for the following reasons. The first one replaces the same Arg103 as in the already characterized R103Q [39] but with Trp rather than with Gln, and it was interesting to compare the two. As recorded in the NCBI dbSNP database (www.ncbi.nlm.nih.gov/snp/rs8191612; accessed on 15 December 2021), the frequency of the germline R103W allele (NC_000008.11:g.11779766C>T) in the general population reaches its maximum (~2.3%) in the groups of the African ancestry, whereas the R103Q allele (NC_000008.11:g.11779767G>A) occurs with high frequency (up to 24%) in East Asian populations. In the homology models of hNEIL2 that were built based on the X-ray structure of mvNEIL2/3 and oNEIL2, Arg103 lies in the large disordered loop within the N-terminal domain of the protein (Figure 1b,c; Supplementary Materials Figure S1). Pro304 was chosen for structural reasons: it is located in the turn of the β_2_ zinc finger (Figure 1b,c; Supplementary Materials Figure S1), a critical DNA-binding element in NEIL2 [38]. The tip of the zinc finger is poorly ordered in oNEIL2 and in the homology models (Supplementary Materials Figure S1), and Pro304 is located right at the edge of the disordered peptide, so its replacement with the less geometrically restrained Thr might be expected to further destabilize the tip of the finger. The allele encoding the P304T variant (NC_000008.11:g.11786184C>A) is most often recorded (~2.2%) from the populations of East Asian origin (www.ncbi.nlm.nih.gov/snp/rs8191666; accessed on 15 December 2021).

### 2.2. Activity of hNEIL2 R103W and P304T on Duplex DNA Substrates

We have overproduced and purified the wild-type (WT) hNEIL2, as well as the R103W and P304T variants, and analyzed their ability to cleave a fluorescently labeled 23-mer DNA duplex containing a single OHU residue opposite G (23OHU//23comp, Table 1) as a standard substrate.

**Figure 3:**
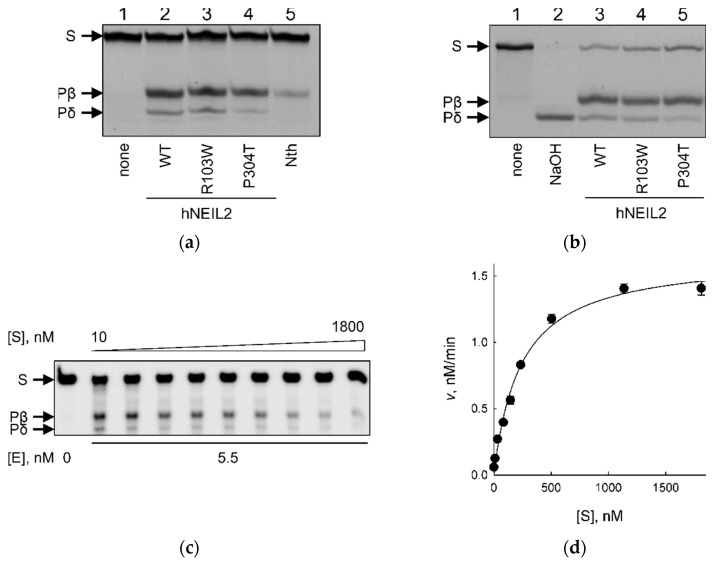
Substrate cleavage by hNEIL2. Cleavage of fluorescently labeled (**a**) OHU:G-containing DNA duplex and (**b**) AP:G-containing DNA duplex (100 nM) by hNEIL2 WT, R103W, and P304T (50 nM each). S, substrate; Pβ, β-elimination product; Pδ, δ-elimination product. (**c**) Representative gel showing the cleavage of varying concentrations of the OHU:G substrate (10–1800 nM, including 10 nM radioactively labeled substrate in each reaction) by hNEIL2 P304T (5.5 nM). (**d**) Plot of reaction velocity vs substrate concentration for the cleavage of 23OHU-containing DNA duplex by hNEIL2 P304T. Mean ± s.e.m. is shown (*n* = 3).

All three of the protein variants were able to cleave the OHU:G substrate (Figure 3a). The isolated AP lyase activity catalyzing β-elimination at a pre-formed AP site, characteristic of bifunctional DNA glycosylases, was also observed for all three of the variants (Figure 3b). H2TH DNA glycosylases are known to catalyze the following three consecutive reactions: hydrolysis of the *N*-glycosidic bond, elimination of the 3′-phosphate group of the nascent abasic site (β-elimination), and elimination of its 5′-phosphate (δ-elimination) [45,46]. In all cases, the product of hNEIL2 cleavage migrated in polyacrylamide gels as two bands. The mobility of the upper one corresponded to the product of OHU cleavage by *E. coli* endonuclease III (Nth), which catalyzes β-elimination, while the lower band co-migrated with the product of NaOH-induced δ-elimination (Figure 3a,b). Thus, both hNEIL2 R103W and P304T share the same basic reaction chemistry with WT hNEIL2.

For a quantitative comparison between hNEIL2 variants, we have determined steady-state kinetic parameters for WT, R103W, and P304T enzymes (Figure 3c,d, Table 2). The *K*_M_ value for hNEIL2 R103W was ~6-fold higher, and for hNEIL2 P304T was ~30-fold higher than that of the wild-type enzyme. The effect of the mutations on *K*_M_ was partially offset by faster enzyme turnover, which was ~5–6-fold higher for both polymorphic variants than that of WT hNEIL2. Such mutually compensatory changes in *k*_cat_ and *K*_M_ are well documented in many enzymatic systems and arise mainly from differences in non-productive substrate binding and/or affinity for the reaction product [47,48,49,50]. Overall, the enzyme efficiency (in terms of *k*_cat_/*K*_M_, or the specificity constant) was close for WT and R103W hNEIL2, and 4–5-fold lower for the P304T variant.

### 2.3. hNEIL2 R103W and P304T Show Reduced Binding to DNA

**Figure 4:**
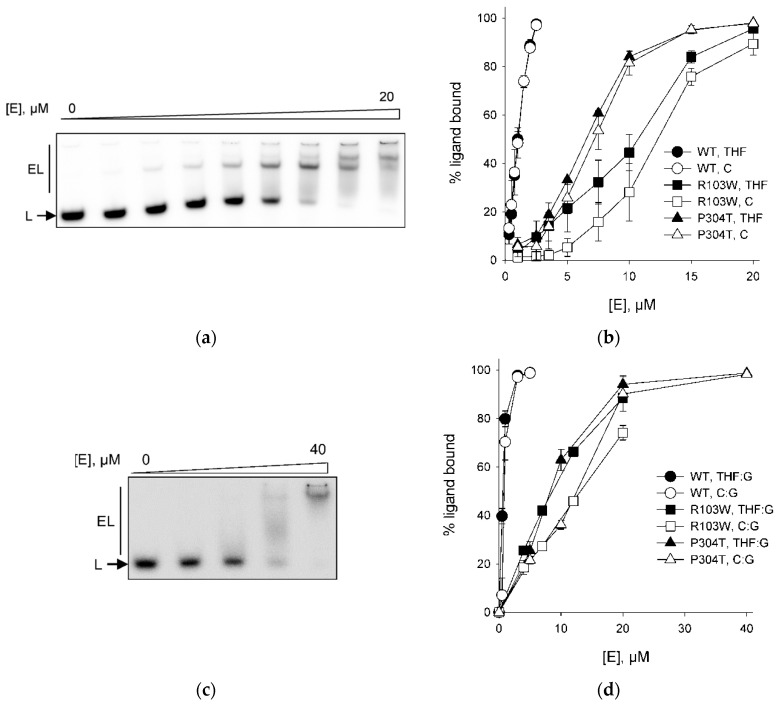
Binding of normal and damaged DNA by hNEIL2. (**a**) Representative gel showing shifting of THF-containing bubble DNA ligand by hNEIL2 P304T (0–20 μM). L, THF bubble ligand; EL, enzyme–ligand complexes. (**b**) Plot of fractional THF bubble binding vs enzyme concentration. Mean ± s.e.m. is shown (*n* = 3). (**c**) Representative gel showing shifting of THF:G-containing DNA duplex by hNEIL2 P304T (0–40 μM). L, THF:G ligand; EL, enzyme–ligand complexes. (**d**) Plot of fractional THF:G binding vs enzyme concentration. Mean ± s.e.m. is shown (*n* = 3).

In order to analyze the DNA-binding properties of hNEIL2 variants, we employed the electrophoretic mobility shift assay [51]. In order to avoid DNA cleavage in the course of the experiment, we used a ^32^P-labeled substrate bearing (3-hydroxytetrahydrofuran-2-yl)methyl phosphate (THF), an AP site analog resistant to β- and β,δ-elimination, as a lesion. NEIL proteins from different species are known to bind THF-containing substrates [52,53,54]. For comparison, we also assessed hNEIL2 variants binding to undamaged DNA containing C instead of a lesion. Since hNEIL2 prefers lesions in DNA bubbles as substrates, we designed a 40-mer ligand containing two 15-bp perfect duplex arms and a centrally located 10-nt bubble with or without THF in the bubble (40F//40bubble or 40C//40bubble, Table 1). With both damaged and normal DNA, the band corresponding to the free ligand disappeared upon increasing protein concentrations, and a slower-migrating band appeared (Figure 4a,b). At higher hNEIL2 concentrations, a second shifted band with even lower mobility was observed for both the undamaged and damaged DNA with all three types of the enzyme (Figure 4a), likely corresponding to non-specific binding of a second protein molecule. Some of the radioactivity was also observed in a smear shifted upwards, indicating partial dissociation of the hNEIL2–DNA complex during its migration through the gel. The apparent binding constants for the wild-type and the mutant hNEIL2, calculated from the fraction combined bound species, are listed in Table 3. Clearly, both R103W and P304T mutants showed a reduced ability to bind DNA bubbles compared with the wild-type protein, with R103W apparently affected to a higher degree. Interestingly, the *K*_bind_^app^ values for THF- and C-containing bubbles were similar, suggesting that hNEIL2 may have a general affinity for bubbles independently of the presence of lesions within them.

We have also assessed the affinity of the wild-type, R103W, and P304T hNEIL2 for double-stranded DNA, either undamaged or containing THF opposite to G. With both of the DNA ligands, we could not observe clear shifted bands; rather, the radiolabeled material shifted upwards in a smear (Figure 4c). The lack of a clear band corresponding to the protein–DNA complex makes it impossible to calculate binding constants, but the overall shapes of the binding curves (Figure 4d) suggest that the binding of hNEIL2 R103W and hNEIL2 P304T, to both normal and damaged double-stranded DNA, is reduced compared to the wild-type protein. Unlike with the bubble DNA, both of the mutants demonstrated the same degree of impairment of full duplex binding. All of the hNEIL2 variants bound the damaged duplex better than the undamaged one, confirming the specific nature of interactions with THF:G-containing DNA.

### 2.4. Activity of hNEIL2 R103W and P304T on Bubble DNA Substrates

**Figure 5:**
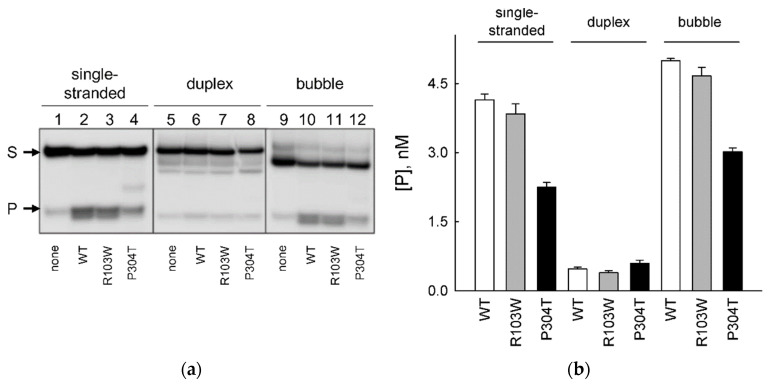
Cleavage of single-stranded, duplex and bubble DHU-containing substrates (15 nM) by hNEIL2 WT, R103W, and P304T (10 nM each). (**a**), Representative cleavage gels. S, substrate; P, combined products. (**b**) Plots of product accumulation; 30 min, 37 °C; enzyme and substrate concentrations as in (**a**). Mean ± s.e.m. is shown (*n* = 3).

Unlike all other DNA glycosylases, NEIL2 shows unique preference for damaged bases within DNA bubbles, 6–18 nt being the optimal bubble size for the enzyme’s activity [11,12]. In order to address the effect of hNEIL2 polymorphisms on its ability to cleave bubbles at damaged sites, we have used 60-mer substrates containing a DHU residue in single-stranded DNA, double-stranded DNA, or in the middle of a 12-nucleotide bubble (Table 1). DHU was chosen over OHU for better stability during synthesis of long oligonucleotides; hNEIL2 prefers OHU in duplexes but cleaves both DHU and OHU with comparable efficiencies in single-stranded DNA and in bubbles [8,11,14]. Figure 5 illustrates the cleavage of these three substrates by WT hNEIL2 and the R103W and P304T variants. Consistent with the reports in the literature [11,12], all of the hNEIL2 variants showed poor activity on DHU in the long duplex substrate (<5% cleavage; Figure 5; also compares with the cleavage of OHU in the duplex in a shorter substrate in Figure 3). With the single-stranded substrate, the activity of WT hNEIL2 and hNEIL2 R103W were similar, whereas the P304T variant showed about two-fold lower cleavage. If the lesion was located in a bubble, it again was processed by WT hNEIL2 and hNEIL2 R103W with similar efficiency, ~20% better than the single-stranded substrate, while the activity of hNEIL2 P304T was ~30–35% lower compared with WT and R103W variants (Figure 5). For WT hNEIL2, the moderate preference of the bubble over the single-stranded substrate was again consistent with the literature [11,12]. Overall, our data indicate that the activity of hNEIL2 P304T is also reduced in single-stranded and bubble DNA substrates.

## 3. Discussion

In this study, we have characterized the biochemical properties of two polymorphic variants of human DNA glycosylase NEIL2, R103W, and P304T. Arg103 lies in the long unordered insert in the N-terminal domain, unique for NEIL2 among other H2TH DNA glycosylases, while Pro304 resides in the β-turn connecting two β-strands of the protein’s C-terminal zinc finger. Overall, the activity of hNEIL2 R103W was on a par with the wild-type enzyme, whereas P304T had lower activity on all of the types of tested substrates.

Before this study, only two natural variants of hNEIL2, R103Q and R257L, were characterized biochemically, both selected for their occurrence in human tumors. Both showed close to normal activity when assayed in isolation, but R257L was deficient in its ability to support the full BER cycle and reduced ability to co-immunoprecitpitate with DNA polymerase β, DNA ligase IIIα, and polynucleotide kinase/3′-phosphatase, which constitute the BER complex [39]. Our results with R103W agree with the absence of the effect of the R103Q mutation. We have noticed that R103W shows reduced affinity for DNA (Figure 4b,d) but this did not immediately aggravate the catalysis, possibly because the affinity for the reaction product was also reduced, leading to an increase in the apparent *k*_cat_ value. The phenomenon of strong product inhibition was reported for many DNA glycosylases (reviewed in [55]) and may be important for protecting the nascent AP site or a DNA break until the assembly of a repair-proficient complex.

The effect of the P304T substitution is easier to interpret on structural grounds. Unlike many sequence-specific DNA-binding proteins that recognize the target through several zinc finger units, the H2TH superfamily DNA glycosylases possess a single Zn^2+^-stabilized β-hairpin of the Cys_4_ or Cys_3_His type, which contacts DNA in the major groove non-specifically through an absolutely conserved Arg residue (Arg310 in hNEIL2) that squeezes two phosphates flanking the lesion [46]. In some cases, such as NEIL1 or plant/fungal MMH proteins, the β-hairpin maintains its shape in the absence of Zn^2+^-coordinating residues (so-called “zincless finger”). The turn connecting two β-strands is fully, or partly, disordered in many of the structures of free H2TH enzymes [15,52,56,57,58,59], including oNEIL2 [14], but becomes well resolved in DNA-bound structures, indicating its intrinsic flexibility, possibly serving to accommodate variable groove geometry. The recent hydrogen/deuterium exchange measurements reveal high exposure of the β-turn to solution in free hNEIL2 [16]. In oNEIL2, Pro307, which is homologous to Pro304, is found immediately adjacent to the disordered tip of the zinc finger [14]. Thus, the location of Pro304 in the structure of hNEIL2 seem to be critical for the correct conformation and dynamics of the zinc finger, and its replacement with conformationally relaxed Thr could affect the protein’s ability to interact with DNA phosphates.

What could be the biological consequences of R103W and P304T mutations? By definition, polymorphisms that are maintained in the general population are either nearly neutral or provide some selective advance to compensate for a functional defect. R103W is known only as a population variant, albeit the change of Arg103 to Gln is recorded in the COSMIC database as a somatic mutation in breast carcinoma (COSV52706850). The P304T variant, in addition to being found in the general population, was found as a somatic mutation in acute myeloid leukemia (COSV52707838). Moreover, a P304S substitution was reported from malignant melanoma (COSV105120005 [60]), and an E305K mutation in an adjacent position, from gastric adenocarcinoma (COSV52706672). In all cases, it is unclear whether these mutations can act as cancer drivers. Based on the little apparent functional difference between WT hNEIL2, hNEIL2 R103Q [39], and hNEIL2 R103W described here, one can expect that the mutations in the position 103 are of no concern. On the other hand, mutations of Pro304 might be a mild cancer risk factor. A useful comparison can be drawn with another human DNA glycosylase, OGG1: its extensively studied S326C allele (rs1052133), frequently occurring in East Asian populations, is an established risk modifier for lung and possibly gastrointestinal cancer but shows only a moderate functional impairment [61,62,63]. Given the total number of carriers (allele frequency ~2.2% in East Asian populations), studies of cancer risk associated with the P304T variant could be warranted.

## 4. Materials and Methods

### 4.1. Enzymes and Oligonucleotides

Bacteriophage T4 polynucleotide kinase was purchased from Biosan (Novosibirsk, Russia), and *E. coli* uracil–DNA glycosylase (Ung), from New England Biolabs (Ipswich, MA, USA). *E. coli* Nth was purified as described [64]. Oligonucleotides (Table 1) were synthesized in-house from commercially available phosphoramidites (Glen Research, Sterling, VA, USA). If necessary, the oligonucleotides either carried a fluorescein label, or were radioactively labeled using γ[^32^P]ATP (SB RAS ICBFM Laboratory of Biotechnology, Novosibirsk, Russia) and phage T4 polynucleotide kinase, as specified in the text. The ^32^P-labeled oligonucleotides were purified by reverse-phase chromatography on an Isolute C18 sorbent (Biotage, Uppsala, Sweden). The AP site-containing substrate was obtained by treating 1 μM duplex 23U//23C (Table 1) with Ung (1 U/μL) in the hNEIL2 reaction buffer (see below) at 37 °C for 15 min and used immediately.

### 4.2. hNEIL2 Purification

pET-21b plasmid carrying a *hNEIL2* insert was kindly provided by Dr. Murat Saparbaev (Paris-Saclay University). The insert was PCR-amplified and re-cloned into pLATE31 using the aLICator ligation-independent cloning kit (Thermo Fisher Scientific, Waltham, MA, USA) according to the manufacturer’s protocol. The resulting construct codes for a full-length hNEIL2 equipped with a C-terminal -GHHHHHH peptide. Site-directed mutagenesis was carried out using the QuikChange Site-Directed Mutagenesis Kit (Agilent Technologies, Santa Clara, CA, USA). The sequences of the inserts were confirmed by Sanger sequencing. *E. coli* BL21(DE3) cells carrying the plasmids were grown overnight at 37 °C with vigorous shaking in 3 mL of LB broth containing 50 μg/mL carbenicillin, inoculated into 50 mL of the same medium, and the growth continued until A_595_ = 0.8. Synthesis of the recombinant protein was induced by adding isopropyl β-D-1-thiogalactopyranoside to 1.5 mM and ZnSO_4_ to 10 μM for 5 h. The bacteria from several cultures grown in parallel were pelleted by centrifugation (12,000× *g* at 4 °C for 30 min), and the cell paste (~12 g) was frozen at −70 °C until further use. For purification, the thawed pellet was resuspended in 20 mM sodium phosphate buffer (pH 7.4) supplemented with 1 mM phenylmethylsulfonyl fluoride and lysed by ultrasound (Q500 sonicator, Qsonica, Newtown, CT, USA). The lysate was clarified by centrifugation (10,000× *g* at 4 °C for 1 h) and loaded on a 5 mL SP-Sepharose column (GE Healthcare, Chicago, IL, USA) equilibrated in 20 mM sodium phosphate (pH 7.4). The proteins were eluted with a 0–1000 mM NaCl gradient in the same buffer and analyzed by 12% polyacrylamide gel electrophoresis (Laemmli system). The fractions containing the protein band with the expected mobility were loaded on a 5 mL Ni–NTA agarose column (GE Healthcare) equilibrated in 20 mM sodium phosphate (pH 7.4) supplemented with 500 mM NaCl and 5 mM imidazole. The proteins were eluted with a 5–500 mM NaCl gradient in the same buffer. The fractions containing the target protein were collected, dialyzed against the storage buffer (50 mM Tris–HCl pH 7.5, 400 mM NaCl, 1 mM EDTA, 1 mM DTT, 50% glycerol) and kept at −20 °C.

### 4.3. Enzyme Activity and Kinetics Measurements

The reaction mixture included 25 mM potassium phosphate (pH 7.5), 5 mM MgCl_2_, 1 mM DTT, and 0.5 mg/mL bovine serum albumin. For the enzyme activity experiments, the substrate concentration was 10 nM or 100 nM, and the enzyme concentration and incubation time was varied as needed. The reactions were allowed to proceed at 37 °C, 10 μL aliquots were withdrawn and terminated by mixing with an equal volume of gel loading solution (20 mM Na-EDTA, 0.1% xylene cyanol, 0.1% bromophenol blue in deionized formamide) and heating for 2 min at 95 °C. For the kinetic experiments, the substrate concentration was 10–1800 nM, of which radioactively labeled substrate constituted 10 nM, and the enzyme concentrations were 1.5 nM for WT hNEIL2, 0.75 nM for hNEIL2 R103W, and 5.5 nM for hNEIL2 P304T. The reactions were allowed to proceed for 60 min at 37 °C and terminated as above. The reaction products were separated by electrophoresis in 20% polyacrylamide/7.2 M urea, and the gels were imaged and quantified using the Typhoon FLA 9500 imager (GE Healthcare).

### 4.4. Electrophoretic Mobiliy Shift Assay

The reaction mixture included 25 mM potassium phosphate (pH 7.5), 5 mM MgCl_2_, 1 mM DTT, 0.5 mg/mL bovine serum albumin, 10% glycerol, 35 nM bubble DNA ligand or 100 nM duplex oligonucleotide ligand, and varying enzyme concentrations. The mixtures were incubated on ice for 5 min and loaded on an 8% non-denaturing polyacrylamide gel. The gels were run at 10 V/cm, thermostated at 4 °C. The gels were imaged and quantified as above. The apparent binding constants were obtained by fitting to a sigmoidal binding model using SigmaPlot v11.0 (Systat Software, Frankfurt am Main, Germany).

### 4.5. In Silico Analysis of the Polymorphism Effect

To predict the possible effects of amino acid changes in hNEIL2, we have used online versions of the following five classifiers: FATHMM [40] (http://fathmm.biocompute.org.uk/, “Inherited disease” prediction mode; all URLs accessed on 15 December 2021), Mutation Assessor [41] (http://mutationassessor.org/r3/, accessed on 15 December 2021), MutationTaster [42] (https://www.mutationtaster.org/, accessed on 15 December 2021), PolyPhen-2 [43] (http://genetics.bwh.harvard.edu/pph2/, accessed on 15 December 2021), and Provean [44] (http://provean.jcvi.org/, “PROVEAN Protein” tool, accessed on 15 December 2021). The output from Mutation Assessor was converted to the binary output by classifying the “neutral/low” prediction as “no effect” (numerical value 0) and the “medium/high” prediction as “affected” (numerical value 1). The binary outputs “tolerated”, “polymorphism” or “neutral” from other algorithms were classified as “no effect”, and “damaging”, “disease causing” or “deleterious” as “affected”. The mean of all predictions was taken as an overall measure of the effect.

### 4.6. Homology Modeling

The homology models of hNEIL2 were built using SwissModel suite [65]. The structures of oNEIL2 (6VJI [14]) and mvNEIL2/3 (4MB7 [15]) served as templates for the open and closed conformations, respectively. Per-residue QMEANDisCo score [66] was used as a local model confidence parameter (Supplementary Materials Figure S1); residues with a score above 0.6 are usually regarded as reliably built in the model.

## Figures and Tables

**Table 1:** Oligonucleotides used in this study.

ID	Sequence, 5′→3′	Modification (X)
23OHU	CTCTCCCTTCXCTCCTTTCCTCT	OHU
23U	CTCTCCCTTCXCTCCTTTCCTCT	U
23F	CTCTCCCTTCXCTCCTTTCCTCT	THF
23C	CTCTCCCTTCCCTCCTTTCCTCT	
23comp	AGAGGAAAGGAGGGAAGGGAGAG	
40C	CCTGCATGGGCGGCAGAACTCGAGGCCATCCTCACCATCC	
40F	CCTGCATGGGCGGCAGAACTXGAGGCCATCCTCACCATCC	THF
40bubble	GGATGGTGAGGATGGGAGGTTCCCTTGCCGCCCATGCAGG	
60DHU	GTAACAGTTCCTGCATGGGCGGCATGAACXGGAGGCCCATCCTCACCATCATCACACTGG	DHU
60comp	CCAGTGTGATGATGGTGAGGATGGGCCTCCGGTTCATGCCGCCCATGCAGGAACTGTTAC	
60bubble	CCAGTGTGATGATGGTGAGGATGGCGAGGTTCCCTCTGCCGCCCATGCAGGAACTGTTAC	

**Table 2:** Kinetic parameters of the cleavage of 23OHU:G-containing DNA duplex by hNEIL2.

Enzyme	*K*_M_, nM	*k*_cat_, min^−1^	*k*_cat_/*K*_M_, μM^−1^·min^−1^
WT	17 ± 2	0.060 ± 0.002	3.5 ± 0.4
R103W	99 ± 9	0.28 ± 0.01	2.8 ± 0.3
P304T	500 ± 30	0.36 ± 0.01	0.72 ± 0.05

**Table 3:** Affinity of hNEIL2 for bubble substrates.

Enzyme	*K*_bind_^app^(THF), μM	*K*_bind_^app^(C), μM
WT	1.0 ± 0.1	1.0 ± 0.1
R103W	10.4 ± 1.2	11.7 ± 0.7
P304T	6.4 ± 0.1	7.0 ± 0.3

## Data Availability

All data are contained in the paper and Supplementary Materials.

## Supplementary Materials

The supporting information can be downloaded at: https://www.mdpi.com/article/10.3390/ijms23042212/s1.

## References

[1] Friedberg E.C., Walker G.C., Siede W., Wood R.D., Schultz R.A., Ellenberger T. (2006). DNA Repair and Mutagenesis.

[2] Beard W.A., Horton J.K., Prasad R., Wilson S.H. (2019). Eukaryotic base excision repair: New approaches shine light on mechanism. Annu. Rev. Biochem..

[3] Caldecott K.W. (2020). Mammalian DNA base excision repair: Dancing in the moonlight. DNA Repair.

[4] Stivers J.T., Jiang Y.L. (2003). A mechanistic perspective on the chemistry of DNA repair glycosylases. Chem. Rev..

[5] Huffman J.L., Sundheim O., Tainer J.A. (2005). DNA base damage recognition and removal: New twists and grooves. Mutat. Res..

[6] Hitomi K., Iwai S., Tainer J.A. (2007). The intricate structural chemistry of base excision repair machinery: Implications for DNA damage recognition, removal, and repair. DNA Repair.

[7] Zharkov D.O. (2008). Base excision DNA repair. Cell. Mol. Life Sci..

[8] Hazra T.K., Kow Y.W., Hatahet Z., Imhoff B., Boldogh I., Mokkapati S.K., Mitra S., Izumi T. (2002). Identification and characterization of a novel human DNA glycosylase for repair of cytosine-derived lesions. J. Biol. Chem..

[9] Hailer M.K., Slade P.G., Martin B.D., Rosenquist T.A., Sugden K.D. (2005). Recognition of the oxidized lesions spiroiminodihydantoin and guanidinohydantoin in DNA by the mammalian base excision repair glycosylases NEIL1 and NEIL2. DNA Repair.

[10] Redrejo-Rodríguez M., Saint-Pierre C., Couve S., Mazouzi A., Ishchenko A.A., Gasparutto D., Saparbaev M. (2011). New insights in the removal of the hydantoins, oxidation product of pyrimidines, via the base excision and nucleotide incision repair pathways. PLoS ONE.

[11] Makasheva K.A., Endutkin A.V., Zharkov D.O. (2020). Requirements for DNA bubble structure for efficient cleavage by helix–two-turn–helix DNA glycosylases. Mutagenesis.

[12] Dou H., Mitra S., Hazra T.K. (2003). Repair of oxidized bases in DNA bubble structures by human DNA glycosylases NEIL1 and NEIL2. J. Biol. Chem..

[13] Zhao X., Krishnamurthy N., Burrows C.J., David S.S. (2010). Mutation versus repair: NEIL1 removal of hydantoin lesions in single-stranded, bulge, bubble, and duplex DNA contexts. Biochemistry.

[14] Eckenroth B.E., Cao V.B., Averill A.M., Dragon J.A., Doublié S. (2021). Unique structural features of mammalian NEIL2 DNA glycosylase prime its activity for diverse DNA substrates and environments. Structure.

[15] Prakash A., Eckenroth B.E., Averill A.M., Imamura K., Wallace S.S., Doublié S. (2013). Structural investigation of a viral ortholog of human NEIL2/3 DNA glycosylases. DNA Repair.

[16] Zhdanova P.V., Ishchenko A.A., Chernonosov A.A., Zharkov D.O., Koval V.V. (2022). Dynamics and conformational changes in human NEIL2 DNA glycosylase analyzed by hydrogen/deuterium exchange mass spectrometry. J. Mol. Biol..

[17] Tate J.G., Bamford S., Jubb H.C., Sondka Z., Beare D.M., Bindal N., Boutselakis H., Cole C.G., Creatore C., Dawson E. (2019). COSMIC: The Catalogue Of Somatic Mutations In Cancer. Nucleic Acids Res..

[18] Chae Y.K., Anker J.F., Carneiro B.A., Chandra S., Kaplan J., Kalyan A., Santa-Maria C.A., Platanias L.C., Giles F.J. (2016). Genomic landscape of DNA repair genes in cancer. Oncotarget.

[19] Osorio A., Milne R.L., Kuchenbaecker K., Vaclová T., Pita G., Alonso R., Peterlongo P., Blanco I., de la Hoya M., Duran M. (2014). DNA glycosylases involved in base excision repair may be associated with cancer risk in *BRCA1* and *BRCA2* mutation carriers. PLoS Genet..

[20] Lamina C., Coassin S., Illig T., Kronenberg F. (2011). Look beyond one’s own nose: Combination of information from publicly available sources reveals an association of *GATA4* polymorphisms with plasma triglycerides. Atherosclerosis.

[21] Benítez-Buelga C., Baquero J.M., Vaclova T., Fernández V., Martín P., Inglada-Perez L., Urioste M., Osorio A., Benítez J. (2017). Genetic variation in the *NEIL2* DNA glycosylase gene is associated with oxidative DNA damage in *BRCA2* mutation carriers. Oncotarget.

[22] Zhai X., Zhao H., Liu Z., Wang L.-E., El-Naggar A.K., Sturgis E.M., Wei Q. (2008). Functional variants of the *NEIL1* and *NEIL2* genes and risk and progression of squamous cell carcinoma of the oral cavity and oropharynx. Clin. Cancer Res..

[23] Koster R., Mitra N., D’Andrea K., Vardhanabhuti S., Chung C.C., Wang Z., Erickson R.L., Vaughn D.J., Litchfield K., Rahman N. (2014). Pathway-based analysis of GWAs data identifies association of sex determination genes with susceptibility to testicular germ cell tumors. Hum. Mol. Genet..

[24] Mou X., Li T., Wang J., Ali Z., Zhang Y., Chen Z., Deng Y., Li S., Su E., Jia Q. (2015). Genetic variation of BCL2 (rs2279115), NEIL2 (rs804270), LTA (rs909253), PSCA (rs2294008) and PLCE1 (rs3765524, rs10509670) genes and their correlation to gastric cancer risk based on universal tagged arrays and Fe_3_O_4_ magnetic nanoparticles. J. Biomed. Nanotechnol..

[25] Ye F., Liu J., Wang H., Chen X., Cheng Q., Chen H. (2020). Cervical carcinoma risk associate with genetic polymorphisms of *NEIL2* gene in Chinese population and its significance as predictive biomarker. Sci. Rep..

[26] Cumova A., Vymetalkova V., Opattova A., Bouskova V., Pardini B., Kopeckova K., Kozevnikovova R., Lickova K., Ambrus M., Vodickova L. (2021). Genetic variations in 3′UTRs of *SMUG1* and *NEIL2* genes modulate breast cancer risk, survival and therapy response. Mutagenesis.

[27] Pardini B., Rosa F., Barone E., Di Gaetano C., Slyskova J., Novotny J., Levy M., Garritano S., Vodickova L., Buchler T. (2013). Variation within 3′-UTRs of base excision repair genes and response to therapy in colorectal cancer patients: A potential modulation of microRNAs binding. Clin. Cancer Res..

[28] He W., Pang L., Gong S., Wang X., Hou L. (2020). Nei endonuclease VIII-like 2 gene rs8191670 polymorphism affects the sensitivity of non-small cell lung cancer to cisplatin by binding with MiR-548a. J. Cancer.

[29] Wei H., Kamat A., Chen M., Ke H.-L., Chang D.W., Yin J., Grossman H.B., Dinney C.P., Wu X. (2012). Association of polymorphisms in oxidative stress genes with clinical outcomes for bladder cancer treated with Bacillus Calmette-Guérin. PLoS ONE.

[30] McGue M., Zhang Y., Miller M.B., Basu S., Vrieze S., Hicks B., Malone S., Oetting W.S., Iacono W.G. (2013). A genome-wide association study of behavioral disinhibition. Behav. Genet..

[31] Kang L., Zou X., Zhang G., Xiang J., Wang Y., Yang M., Chen X., Wu J., Guan H. (2019). A variant in a microRNA binding site in NEIL2 3′UTR confers susceptibility to age-related cataracts. FASEB J..

[32] Kinslow C.J., El-Zein R.A., Hill C.E., Wickliffe J.K., Abdel-Rahman S.Z. (2008). Single nucleotide polymorphisms 5′ upstream the coding region of the *NEIL2* gene influence gene transcription levels and alter levels of genetic damage. Genes Chromosomes Cancer.

[33] Chakraborty A., Wakamiya M., Venkova-Canova T., Pandita R.K., Aguilera-Aguirre L., Sarker A.H., Singh D.K., Hosoki K., Wood T.G., Sharma G. (2015). *Neil2*-null mice accumulate oxidized DNA bases in the transcriptionally active sequences of the genome and are susceptible to innate inflammation. J. Biol. Chem..

[34] Sayed I.M., Sahan A.Z., Venkova T., Chakraborty A., Mukhopadhyay D., Bimczok D., Beswick E.J., Reyes V.E., Pinchuk I., Sahoo D. (2020). *Helicobacter pylori* infection downregulates the DNA glycosylase NEIL2, resulting in increased genome damage and inflammation in gastric epithelial cells. J. Biol. Chem..

[35] Sayed I.M., Chakraborty A., Abd El-Hafeez A.A., Sharma A., Sahan A.Z., Huang W.J.M., Sahoo D., Ghosh P., Hazra T.K., Das S. (2020). The DNA glycosylase NEIL2 suppresses *Fusobacterium*-infection-induced inflammation and DNA damage in colonic epithelial cells. Cells.

[36] Englander E.W., Ma H. (2006). Differential modulation of base excision repair activities during brain ontogeny: Implications for repair of transcribed DNA. Mech. Ageing Dev..

[37] Bhakat K.K., Hazra T.K., Mitra S. (2004). Acetylation of the human DNA glycosylase NEIL2 and inhibition of its activity. Nucleic Acids Res..

[38] Das A., Rajagopalan L., Mathura V.S., Rigby S.J., Mitra S., Hazra T.K. (2004). Identification of a zinc finger domain in the human NEIL2 (Nei-like-2) protein. J. Biol. Chem..

[39] Dey S., Maiti A.K., Hegde M.L., Hegde P.M., Boldogh I., Sarkar P.S., Abdel-Rahman S.Z., Sarker A.H., Hang B., Xie J. (2012). Increased risk of lung cancer associated with a functionally impaired polymorphic variant of the human DNA glycosylase NEIL2. DNA Repair.

[40] Shihab H.A., Gough J., Cooper D.N., Stenson P.D., Barker G.L.A., Edwards K.J., Day I.N.M., Gaunt T.R. (2013). Predicting the functional, molecular, and phenotypic consequences of amino acid substitutions using hidden Markov models. Hum. Mutat..

[41] Reva B., Antipin Y., Sander C. (2011). Predicting the functional impact of protein mutations: Application to cancer genomics. Nucleic Acids Res..

[42] Schwarz J.M., Cooper D.N., Schuelke M., Seelow D. (2014). MutationTaster2: Mutation prediction for the deep-sequencing age. Nat. Methods.

[43] Adzhubei I.A., Schmidt S., Peshkin L., Ramensky V.E., Gerasimova A., Bork P., Kondrashov A.S., Sunyaev S.R. (2010). A method and server for predicting damaging missense mutations. Nat. Methods.

[44] Choi Y., Sims G.E., Murphy S., Miller J.R., Chan A.P. (2012). Predicting the functional effect of amino acid substitutions and indels. PLoS ONE.

[45] Dodson M.L., Michaels M.L., Lloyd R.S. (1994). Unified catalytic mechanism for DNA glycosylases. J. Biol. Chem..

[46] Zharkov D.O., Shoham G., Grollman A.P. (2003). Structural characterization of the Fpg family of DNA glycosylases. DNA Repair.

[47] Brot F.E., Bender M.L. (1969). Use of the specificity constant of α-chymotrypsin. J. Am. Chem. Soc..

[48] Fastrez J., Fersht A.R. (1973). Mechanism of chymotrypsin. Structure, reactivity, and nonproductive binding relations. Biochemistry.

[49] Szeltner Z., Rea D., Juhász T., Renner V., Mucsi Z., Orosz G., Fülöp V., Polgár L. (2002). Substrate-dependent competency of the catalytic triad of prolyl oligopeptidase. J. Biol. Chem..

[50] Yu B., Hunt J.F. (2009). Enzymological and structural studies of the mechanism of promiscuous substrate recognition by the oxidative DNA repair enzyme AlkB. Proc. Natl Acad. Sci. USA.

[51] Smider V., Hwang B.J., Chu G. (2006). Electrophoretic mobility shift assays to study protein binding to damaged DNA. Methods Mol. Biol..

[52] Imamura K., Wallace S.S., Doublié S. (2009). Structural characterization of a viral NEIL1 ortholog unliganded and bound to abasic site-containing DNA. J. Biol. Chem..

[53] Grin I.R., Rieger R.A., Zharkov D.O. (2010). Inactivation of NEIL2 DNA glycosylase by pyridoxal phosphate reveals a loop important for substrate binding. Biochem. Biophys. Res. Commun..

[54] Zhu C., Lu L., Zhang J., Yue Z., Song J., Zong S., Liu M., Stovicek O., Gao Y.Q., Yi C. (2016). Tautomerization-dependent recognition and excision of oxidation damage in base-excision DNA repair. Proc. Natl Acad. Sci. USA.

[55] Endutkin A.V., Yudkina A.V., Sidorenko V.S., Zharkov D.O. (2019). Transient protein–protein complexes in base excision repair. J. Biomol. Struct. Dyn..

[56] Golan G., Zharkov D.O., Feinberg H., Fernandes A.S., Zaika E.I., Kycia J.H., Grollman A.P., Shoham G. (2005). Structure of the uncomplexed DNA repair enzyme endonuclease VIII indicates significant interdomain flexibility. Nucleic Acids Res..

[57] Duclos S., Aller P., Jaruga P., Dizdaroglu M., Wallace S.S., Doublié S. (2012). Structural and biochemical studies of a plant formamidopyrimidine-DNA glycosylase reveal why eukaryotic Fpg glycosylases do not excise 8-oxoguanine. DNA Repair.

[58] Liu M., Imamura K., Averill A.M., Wallace S.S., Doublié S. (2013). Structural characterization of a mouse ortholog of human NEIL3 with a marked preference for single-stranded DNA. Structure.

[59] Prakash A., Carroll B.L., Sweasy J.B., Wallace S.S., Doublié S. (2014). Genome and cancer single nucleotide polymorphisms of the human NEIL1 DNA glycosylase: Activity, structure, and the effect of editing. DNA Repair.

[60] Rabbie R., Ferguson P., Wong K., Couturier D.-L., Moran U., Turner C., Emanuel P., Haas K., Saunus J.M., Davidson M.R. (2021). The mutational landscape of melanoma brain metastases presenting as the first visceral site of recurrence. Br. J. Cancer.

[61] Dherin C., Radicella J.P., Dizdaroglu M., Boiteux S. (1999). Excision of oxidatively damaged DNA bases by the human α-hOgg1 protein and the polymorphic α-hOgg1(Ser326Cys) protein which is frequently found in human populations. Nucleic Acids Res..

[62] Simonelli V., Camerini S., Mazzei F., Van Loon B., Allione A., D’Errico M., Barone F., Minoprio A., Ricceri F., Guarrera S. (2013). Genotype–phenotype analysis of S326C OGG1 polymorphism: A risk factor for oxidative pathologies. Free Radic. Biol. Med..

[63] Kang S.W., Kim S.K., Park H.J., Chung J.-H., Ban J.Y. (2017). Human 8-oxoguanine DNA glycosylase gene polymorphism (Ser326Cys) and cancer risk: Updated meta-analysis. Oncotarget.

[64] Kuznetsov N.A., Kladova O.A., Kuznetsova A.A., Ishchenko A.A., Saparbaev M.K., Zharkov D.O., Fedorova O.S. (2015). Conformational dynamics of DNA repair by *Escherichia coli* endonuclease III. J. Biol. Chem..

[65] Waterhouse A., Bertoni M., Bienert S., Studer G., Tauriello G., Gumienny R., Heer F.T., de Beer T.A.P., Rempfer C., Bordoli L. (2018). SWISS-MODEL: Homology modelling of protein structures and complexes. Nucleic Acids Res..

[66] Studer G., Rempfer C., Waterhouse A.M., Gumienny R., Haas J., Schwede T. (2020). QMEANDisCo—Distance constraints applied on model quality estimation. Bioinformatics.

