# Supplementary material for: A Low-Activity Polymorphic Variant of Human NEIL2 DNA Glycosylase
Source: Int J Mol Sci. 2022 Feb 17;23(4):2212. doi: 10.3390/ijms23042212 (PMC8879280; doi:10.3390/ijms23042212)
Supplement: Supplementary file 1 [file ijms-23-02212-s001.zip › Figure S1.pdf]

Supporting Information for

Kakhkharova *et al.*, A low-activity polymorphic variant of human NEIL2 DNA glycosylase

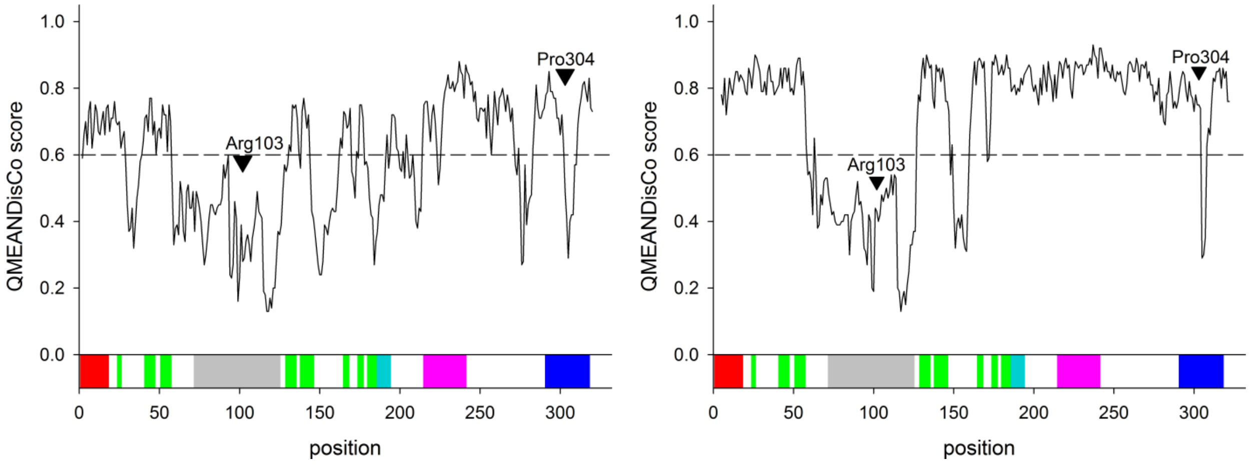

(a) **(b)**

Fig. S1. Per-residue QMEANDisCo score plotted against the residue number for the homology model of hNEIL2 in the closed conformation based on the mvNEIL2/3 template (a) and the homology model of hNEIL2 in the open conformation based on the oNEIL2 template (b). The colored bar below corresponds to the main elements of hNEIL2 structure as inferred based on sequence homology with oNEIL2 and mvNEIL2/3: red, the N-terminal α-helix carrying the catalytic Pro and Glu; green, β-sandwich in the N-terminal domain; grey, the disordered loop; turquoise, the interdomain linker; magenta, the H2TH motif; blue, the zinc finger. Residues with a QMEANDisCo score above 0.6 are usually regarded as reliably built in the model.
