# Supplementary material for: A Low-Activity Polymorphic Variant of Human NEIL2 DNA Glycosylase
Source: Int J Mol Sci. 2022 Feb 17;23(4):2212. doi: 10.3390/ijms23042212 (PMC8879280; doi:10.3390/ijms23042212)
Supplement: Supplementary file 1 [file ijms-23-02212-s001.zip › Figure S2.pdf]

Supporting Information for

Kakhkharova *et al.*, A low-activity polymorphic variant of human NEIL2 DNA glycosylase

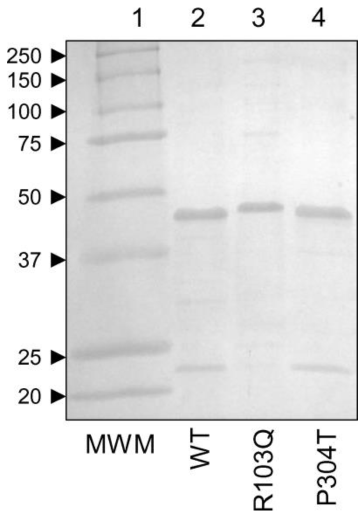

Fig. S2. SDS-polyacrylamide gel stained with InstantBlue Ultrafast Protein Stain (Sigma-Aldrich) showing the preparations of hNEIL2 proteins (lane 2, wild-type; lane 3, R103Q; lane 4, P304T). MWM, molecular weight markers; the weights are indicated by arrowheads.
